# Trace Amine-Associated Receptor Type 1 as A Target for The Development of Treatments for Stimulant Abuse

**DOI:** 10.4172/2329-6488.1000e122

**Published:** 2015-06-12

**Authors:** Takato Hiranita, David A. Thorn

**Affiliations:** Division of Neurotoxicology, National Center for Toxicological Research, U.S. Food and Drug Administration, 3900 NCTR Road, Jefferson, AR 72079-9501, USA

New research has implicated a potential target in the treatment of stimulant abuse. As with opioid abuse, stimulant abuse consists of a primary group for substance abuse disorders. However, there is no medication for the treatment of stimulant abuse such as amphetamines, substrates for the dopamine transporter, and cocaine, a dopamine reuptake inhibitor. Stimulants have in common a capacity to increase extracellular DA levels in terminal regions of mesolimbic dopaminergic neurons [1, 2]. Preclinical studies suggest that dopamine receptor antagonists possess the potential as medications for stimulant abuse while clinical studies failed to support the positive findings in laboratory animals [3]. The negative findings with dopamine receptor antagonists might result from their capacity to produce “surmountable” antagonism of stimulant self-administration since the reinforcing effects of stimulants can be restored in the presence of the dopamine receptor antagonist if doses of stimulants are increased [4, 5].

As with methadone for heroin abuse [6], recent preclinical studies suggest clinically preferential profiles (i.e. “insurmountable” antagonism) of several novel targets as treatments for stimulant abuse [7, 8]. Among them is the trace amine-associated receptors type 1(TAAR1) that possess anti-stimulant actions. For example, Dr. Jun-Xu Li and his co-workers discovered appreciable effectiveness of a TAAR1 agonist in decreasing the abuse-related effects of stimulants in rats. First, the TAAR1 partial agonist RO5263397 [*S*-4-(3-fluoro-2-methylphenyl)-4,5-dihydro-oxazol-2-ylamine] decreased the behavioral sensitizing effects of cocaine and also attenuated the established place-conditioning effects of cocaine [9, 10]. In addition, pretreatment with the TAAR1 partial agonist RO5263397 resulted in decreases in self-administration responding maintained by cocaine injections in a behavioral economics study [9]. Further, RO5263397 shifted down a dose-effect curve of methamphetamine self-administration under a fixed-ratio schedule of reinforcement [8]. In addition, another study from Dr. Canales and his colleagues also demonstrated preclinical efficacy of another TAAR1 partial agonist RO5203648 [*S*-4-(3,4-dichloro-phenyl)-4,5-dihydro-oxazol-2-ylamine] (Figure 1) as cocaine antagonist [11]. Thus, RO5263397 would be effective regardless of doses of stimulant. Unfortunately there was no assessment of dose-dependency and behavioral specificity for the anti-stimulant effects of the TAAR1 agonist; however, the capacity to produce insurmountable antagonism of stimulant self-administration appears to be preferential for medications for stimulant abuse.

In summary, the capacity to produce insurmountable antagonism of stimulant self-administration appears to be a key feature for the development of treatments for stimulant abuse. There are very few such pharmacological targets. However, in efforts to discover novel targets for stimulant abuse, TAAR1 is worthwhile to pursue as a viable target as TAAR1 agonists decrease some of the abuse-related effects of stimulants. Therefore, drugs acting on TAAR1 have potential as pharmacological medications to treat stimulant abuse.

## Figures and Tables

**Figure 1:**
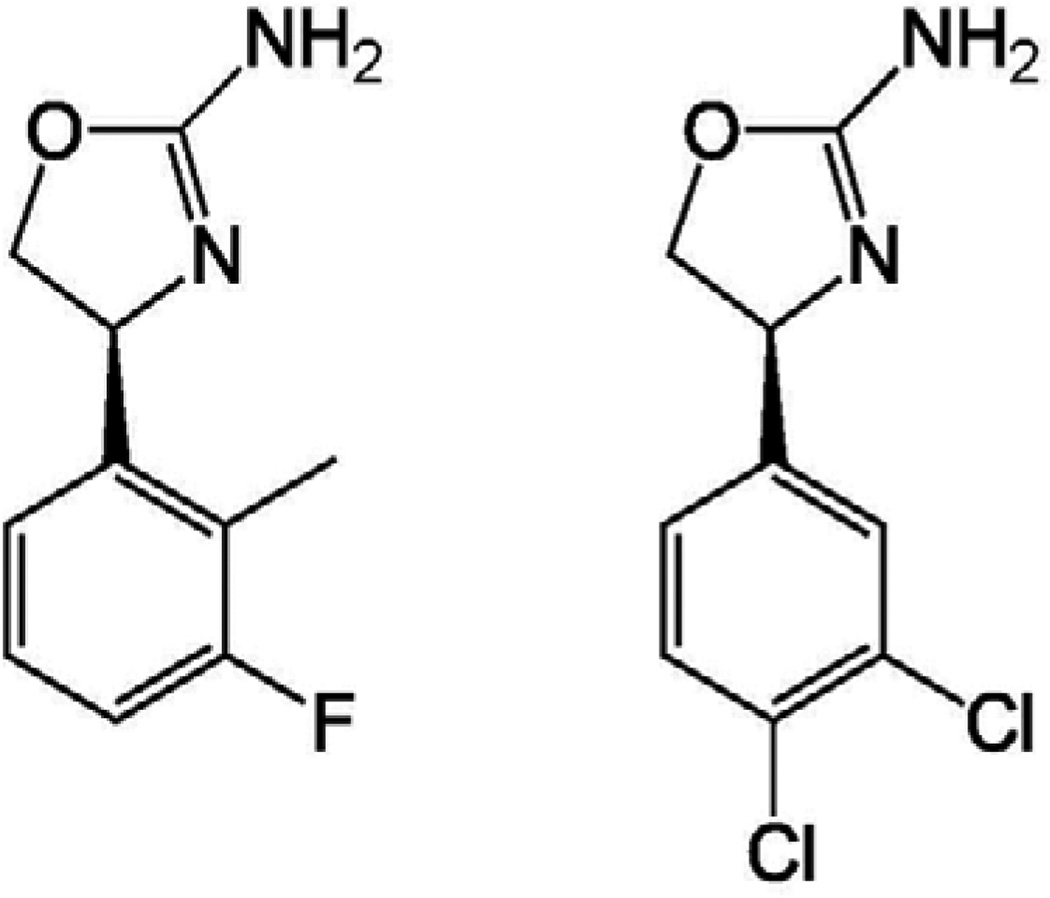
Chemical structures of RO5263397 [S-4-(3-fluoro-2-methylphenyl)-4,5-dihydro-oxazol-2-ylamine, left] and RO5203648 [S-4-(3,4-dichloro-phenyl)-4,5-dihydro-oxazol-2-ylamine, right].
